# The Transdiagnostic Intervention for Sleep and Circadian Dysfunction (TranS-C) for serious mental illness in community mental health part 2: study protocol for a hybrid type 2 effectiveness-implementation cluster-randomized trial using train-the-trainer

**DOI:** 10.1186/s13063-023-07523-6

**Published:** 2023-08-07

**Authors:** Catherine A. Callaway, Laurel D. Sarfan, Emma R. Agnew, Lu Dong, Julia M. Spencer, Rafael Esteva Hache, Marlen Diaz, Shayna A. Howlett, Krista R. Fisher, Heather E. Hilmoe Yates, Eric Stice, Amy M. Kilbourne, Daniel J. Buysse, Allison G. Harvey

**Affiliations:** 1 University of California, Berkeley, CA USA; 2 RAND Corporation, Santa Monica, CA USA; 3 Stanford University, Stanford, CA USA; 4 University of Michigan and VA Ann Arbor Healthcare System, Ann Arbor, MI USA; 5 University of Pittsburgh, Pittsburgh, PA USA

**Keywords:** Train-the-trainer, Transdiagnostic, Sleep, Circadian, Serious mental illness, Implementation, Sustainment, Adaptation, Community mental health

## Abstract

**Background:**

Train-the-trainer (TTT) is a promising method for implementing evidence-based psychological treatments (EBPTs) in community mental health centers (CMHCs). In TTT, expert trainers train locally embedded individuals (i.e., Generation 1 providers) to deliver an EBPT, who then train others (i.e., Generation 2 providers). The present study will evaluate implementation and effectiveness outcomes of an EBPT for sleep and circadian dysfunction—the Transdiagnostic Intervention for Sleep and Circadian Dysfunction (TranS-C)—delivered to CMHC patients with serious mental illness by Generation 2 providers (i.e., trained and supervised within CMHCs via TTT). Specifically, we will investigate whether adapting TranS-C to fit CMHC contexts improves Generation 2 (a) patient outcomes and (b) providers’ perceptions of fit.

**Methods:**

TTT will be implemented in nine CMHCs in California, USA (*N* = 60 providers; *N* = 130 patients) via facilitation. CMHCs are cluster-randomized by county to Adapted TranS-C or Standard TranS-C. Within each CMHC, patients are randomized to immediate TranS-C or usual care followed by delayed treatment with TranS-C (UC-DT). Aim 1 will assess the effectiveness of TranS-C (combined Adapted and Standard), compared to UC-DT, on improvements in sleep and circadian problems, functional impairment, and psychiatric symptoms for Generation 2 patients. Aim 2 will evaluate whether Adapted TranS-C is superior to Standard TranS-C with respect to Generation 2 providers’ perceptions of fit. Aim 3 will evaluate whether Generation 2 providers’ perceived fit mediates the relation between TranS-C treatment condition and patient outcomes. Exploratory analyses will (1) evaluate whether the effectiveness of TranS-C for patient outcomes is moderated by generation, (2) compare Adapted and Standard TranS-C on patient perceptions of credibility/improvement and PhenX Toolkit outcomes (e.g., substance use, suicidality), and (3) evaluate other possible moderators.

**Discussion:**

This trial has potential to (a) inform the process of embedding local trainers and supervisors to expand delivery of a promising transdiagnostic treatment for sleep and circadian dysfunction, (b) add to the growing body of TTT literature by evaluating TTT outcomes with a novel treatment and population, and (c) advance our understanding of providers’ perceptions of EBPT “fit” across TTT generations.

**Trial registration:**

ClinicalTrials.gov identifier NCT05805657. Registered on April 10, 2023.

## Introduction

Although there has been a proliferation in evidence-based psychological treatments (EBPTs), transfer to routine practice settings has been slow [1]. The field of implementation science has taken strides to address a host of variables influencing the use of EBPTs in routine practice settings (e.g., organizational, fiscal, policy) [2]. However, a remaining issue is that many providers, particularly in community mental health care, do not receive sufficient training in EBPTs [3]. Moreover, when providers *are* trained in EBPTs, the methods used for training are not necessarily effective or sustainable [4]. Thus, to expand access to EBPTs, an important next step is to develop and evaluate effective, scalable, and sustainable approaches to EBPT training in community settings.

Review papers over the past two decades have converged on key training elements as essential features of EBPT trainings, namely: a workshop utilizing active learning strategies, a provider manual, and ongoing clinical supervision and/or consultation (e.g., [4, 5]). However, there are significant barriers to widespread implementation of these multicomponent training initiatives in community settings, including insufficient time and funding, shortage of trainers and consultants, staff turnover, and staff resistance to changes in the status quo [6, 7]. Indeed, many sites that initially embraced EBPTs after training have not sustained the practice [8]. Thus, the critical unanswered question is: how can these multicomponent trainings be cost-efficient, acceptable, and sustainable in community settings so that the benefits of EBPTs remain available for the vulnerable populations they serve over the long term?

The present study seeks to examine one possible solution: namely, the train-the-trainer (TTT) approach. Also called a “pyramidal” or “cascading” model, TTT is theorized to be the most promising method of implementing, scaling up, and sustaining training efforts [4, 5]. TTT is a training structure with multiple levels, which we will call “Generations.” First, external “expert trainers” train an initial cohort of providers in a specific EBPT. These providers in the initial cohort are referred to as “Generation 1 providers.” Next, Generation 1 providers are offered additional training on how to *train others* in the EBPT and become “local trainers.” These local trainers then train the next cohort of providers within their organization, who are referred to as “Generation 2 providers.” Local trainers can train future cohorts of providers and/or trainers as needed in response to staff turnover and patient demand. TTT is theorized to (a) be more cost-effective long term relative to the cost of repeated use of an external expert trainer, (b) reduce the impact of provider turnover on EBPT sustainability, and (c) foster an organizational climate and culture that will sustain the EBPT [6].

A fundamental question that TTT studies seek to answer is “when the torch is passed, does the flame still burn?” (p. 726) [9]. In other words, are key outcomes maintained after the transition from Generation 1 to Generation 2? Importantly, results have been mixed with respect to this question when looking across TTT studies for a variety of populations, including post-traumatic stress disorder [10], substance use [11], autism spectrum disorder [12], and individuals at risk for eating disorder [13]. Promisingly, many prior studies show no difference between generations on select outcomes, such as training effectiveness [14], provider competence [11, 15], and patient outcomes [16]. Moreover, in a recent study by Fitzsimmons-Craft and colleagues (2021), there is an encouraging signal that TTT appears to sustain provider adherence and competence to the EBPT better than expert consultation [17]. However, there is also evidence of a scale-up penalty (i.e., poorer outcomes) in Generation 2. Southam-Gerow et al. (2014) found poorer quality case materials were submitted by providers trained by local trainers compared to those of providers trained by national experts [15]. In a recent study conducted by Brabson and colleagues (2021), providers trained in Generation 2 were less knowledgeable about the EBPT and less satisfied with the training compared to those trained in Generation 1 [6]. However, it is important to note that research on TTT is still in its infancy. TTT studies tend to suffer from small sample sizes, brief or no follow-up periods, and lack of methodological rigor [4, 5], and relatively few clinical populations have been investigated. As TTT becomes more commonly used as an implementation and sustainment strategy, more research is needed to ascertain whether key implementation and EBPT outcomes are maintained after training and supervision responsibilities have been passed to locally embedded individuals in Generation 2 (and beyond).

The present trial seeks to continue progressing knowledge in this domain in the context of a transdiagnostic EBPT for sleep and circadian dysfunction—the Transdiagnostic Intervention for Sleep and Circadian Dysfunction (TranS-C)—for serious mental illness (SMI) in community mental health centers (CMHCs) [18]. TranS-C is a skills-based, psychosocial, and modular treatment that was developed in response to mounting evidence that sleep and circadian dysfunction is a transdiagnostic contributor to SMI [19]. In an initial efficacy trial, in which university-based therapists delivered TranS-C to adult CMHC patients, the results were promising. Specifically, relative to usual care followed by delayed treatment with TranS-C (UC-DT), TranS-C was associated with improvements in sleep and circadian problems, functional impairment, and psychiatric symptoms [20].

The present hybrid type 2 effectiveness-implementation trial takes two important strides forward to test TranS-C in CMHCs. First, we will test TranS-C delivered by Generation 2 CMHC providers who are *trained and supervised within CMHCs via TTT*. Second, we will test two versions of TranS-C: Standard TranS-C and Adapted TranS-C. In the initial efficacy trial, CMHC staff identified dose and complexity as barriers to implementing TranS-C (henceforth “Standard TranS-C”) [21]. In response, guided by the Enhanced Replicating Effective Programs framework (REP) [22, 23], our team developed a modified version of TranS-C—henceforth “Adapted TranS-C”—to improve *fit* with the CMHC context (see “Method” and Sarfan et al. (2023) [24] for systematic development of Adapted TranS-C). Importantly, fit predicts a host of implementation outcomes, including reach, treatment fidelity, and sustained use of treatments [25, 26]. Thus, another core focus of the present trial is to determine if “fit,” operationalized herein as provider perceptions of TranS-C acceptability, appropriateness, and feasibility, could be improved in the context of CMHCs.

Following the National Institute of Health stage model [27], the present trial is conducted over the course of three phases to test TranS-C with CMHC providers. In the first phase, the Implementation Phase, we will test implementation and effectiveness outcomes of Standard and Adapted TranS-C with CMHC providers, who are trained by treatment experts (i.e., Generation 1 of TTT) [24]. In the second phase, the Train-the-Trainer Phase and the focus of this protocol, we will test implementation and effectiveness outcomes of Standard and Adapted TranS-C with CMHC providers who are *trained and supervised within CMHCs* (i.e., Generation 2 of TTT). In the third phase, the Sustainment Phase, we will focus on sustainment outcomes (Sarfan et al., in preparation). Importantly, to the best of our knowledge, this is the first study to utilize a TTT approach for sleep treatment for adults with SMI in CMHCs, let alone using this approach with Standard *and* Adapted versions of a transdiagnostic treatment. Together, this trial builds upon recent efforts to tackle the complex challenge of sustaining transdiagnostic, modular treatment approaches in real-world settings [9].

## Aims

This study aims to evaluate the implementation and effectiveness outcomes of TranS-C in the CMHCs of counties across California in the USA. As explained above, the present protocol describes Phase 2, the Train-the-Trainer Phase, of a three-part hybrid type 2 effectiveness-implementation study. The Train-the-Trainer Phase builds upon the infrastructure of the Implementation Phase [24]. Specifically, during the Implementation Phase, sites are cluster-randomized by county to Adapted TranS-C or Standard TranS-C with 1:1 allocation. External expert trainers train an initial cohort of providers (i.e., Generation 1 providers) in TranS-C via facilitation. Then, within each county, patients are randomized to receive immediate TranS-C or UC-DT from Generation 1 providers.

**Fig. 1:**
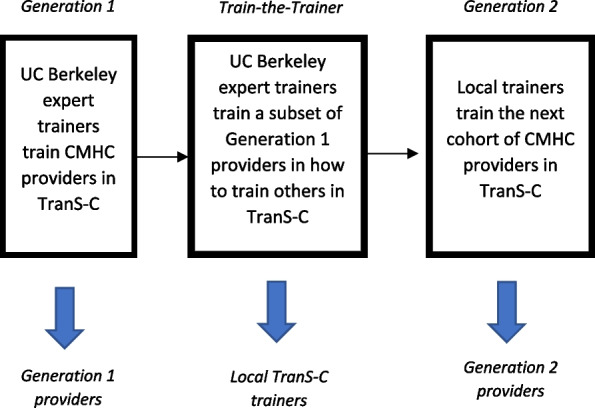
Train-the-Trainer Model

In the Train-the-Trainer Phase, expert trainers offer additional training for Generation 1 providers to become “local trainers” in TranS-C. Then, these local trainers train the next cohort of providers (i.e., Generation 2 providers) within their organization in TranS-C (see Fig. 1). Then, within each county, patients are randomized to receive immediate TranS-C or UC-DT from Generation 2 providers. Patients treated by Generation 2 providers are referred to as “Generation 2 patients.” Sites retain their original randomization assignment to Adapted or Standard TranS-C. See below for more details on the TTT procedures.

Aims and hypotheses for the Train-the-Trainer Phase mirror the Implementation Phase [24]. Parallel analyses to the Implementation Phase will allow us to test the extent to which implementation and effectiveness outcomes are maintained with TTT.

The first aim is to assess the effectiveness of TranS-C, compared to UC-DT, in patients who are treated by Generation 2 providers. We hypothesize that, compared to UC-DT, TranS-C (combined Adapted and Standard) will be associated with larger reductions in the primary patient outcome of sleep disturbance and the secondary patient outcomes of sleep-related impairment, functional impairment, and psychiatric symptoms. We also hypothesize that TranS-C’s benefits for functional impairment and psychiatric symptoms will be mediated by improvements in sleep and circadian problems. The second aim is to evaluate whether TranS-C treatment condition (Adapted vs. Standard TranS-C) is associated with fit to the CMHC context, operationalized as provider ratings of acceptability, appropriateness, and feasibility, for Generation 2 providers. We hypothesize that Adapted TranS-C will be superior to Standard TranS-C with respect to the primary provider outcome of acceptability and the secondary provider outcomes of appropriateness and feasibility. The third aim is to evaluate whether perceived fit among Generation 2 providers mediates the relation between TranS-C treatment condition and Generation 2 patient outcomes. We hypothesize that relative to Standard TranS-C, Adapted TranS-C will be associated with greater reductions in the primary and secondary patient outcomes indirectly through higher provider ratings of acceptability, appropriateness, and feasibility. Exploratory analyses will (1) compare whether the effectiveness of TranS-C for primary and secondary patient outcomes is moderated by generation, (2) compare Adapted and Standard TranS-C on Generation 2 patient perceptions of credibility and perceived improvement and select PhenX Toolkit outcomes that are strongly related to both SMI and sleep and circadian problems (e.g., substance use, suicidality) [28, 29] and of high priority to our community partners, and (3) determine whether treatment effects for Generation 2 patients are moderated by risk factors including age, sex, and sleep and circadian and psychiatric symptoms at baseline. In particular, emerging evidence suggests that patients who are older and have more severe sleep and circadian and psychiatric symptoms at baseline demonstrate poorer response to sleep and circadian treatment, whereas outcomes by sex have been mixed for patients with SMI [30, 31].

## Method

This study was preregistered on ClinicalTrials.gov (identifier: NCT05805657) and received approval from the Committee for the Protection of Human Subjects at the University of California, Berkeley. Any protocol changes will be submitted to ClinicalTrials.gov and the Committee for the Protection of Human Subjects. The research team will communicate relevant changes to the CMHCs and in appropriate publications (e.g., see subsection on “Changes to preregistration” below). If there are too many findings to reasonably interpret in one paper, we may separate some of the findings into two or more papers. This research is funded by the National Institute of Mental Health (R01MH120147). The present protocol used the SPIRIT reporting guidelines [32] (see SPIRIT checklist in supplemental documents and Table 2).

### Train-the-Trainer

Throughout Phases 1 (Implementation) and 2 (Train-the-Trainer), implementation is conducted via facilitation [33]. Specifically, each CMHC receives direct support from a lead facilitator, who is a licensed clinical social worker with expertise in community mental health and sleep treatment (ERA), and a team of trained facilitators employed by the research team. Facilitation is based on the REP framework [23] and was selected as the core implementation strategy used to implement TranS-C in the CMHCs, based on promising evidence [34]. The UC Berkeley facilitation team transitioned CMHC sites to the Train-the-Trainer Phase on a rolling basis. Each site’s readiness for TTT was assessed by the level of provider engagement, the number of patients who had completed sleep treatment, and the supportiveness of leadership. The first site was transitioned to TTT in December 2020, and all sites were transitioned by December 2022. Treatment recruitment will continue through 2023.

In the Train-the-Trainer Phase, the facilitators’ primary activities are (1) recruiting, training, and providing consultation for local trainers and (2) recruiting and enrolling Generation 2 providers and patients. While local trainers were heavily involved in increasing provider adoption and utilization of TranS-C, the facilitators remained in charge of recruiting and enrolling providers and patients through the formal study procedures (e.g., consent, assessments) to reduce burden. Facilitators also hold as-needed consultation for TranS-C providers across generations, offer certification in sleep treatment and sleep training, process Continuing Education credits, and organize regular meetings with CMHC leadership to provide ongoing support and problem-solve barriers in implementing TranS-C. After local trainers hold their first training, the facilitation team gradually transfers select responsibilities to them (e.g., presenting to CMHC providers on advanced sleep-related topics; supervising TranS-C cases on the path to certification), all of which are noted in the “Generation 2” and “Recruitment” sections below. In other words, the role of facilitators shifted from full facilitation [33] toward technical assistance [22] as local trainers gained mastery and independence. A gradual approach was selected to enable facilitators to provide sufficient modeling, support, and feedback for local trainers and transfer responsibility to local trainers at a pace that felt manageable.

#### Training local trainers

Local trainers consist of Generation 1 providers who were trained to deliver TranS-C by the lead facilitator, who is also the “expert trainer,” during the Implementation Phase [24]. To train Generation 1 providers to be local trainers, the expert trainer first led a 30-min welcome meeting to provide an overview of the process and offer training in public speaking. Next, the TranS-C training material was condensed into “big picture” concepts and the content was divided into 1-h chunks. The expert trainer then conducted “booster trainings” for local trainers to review each content chunk (4–5 boosters for Adapted TranS-C, 6–7 boosters for Standard TranS-C). Before each booster training and to facilitate active learning, the expert trainer assigned each local trainer a selection of slides to present. The expert trainer also provided materials to support the trainer to prepare (e.g., a video recording of the expert trainer presenting the same material, a written overview synthesizing the big picture concepts). In between booster trainings, the expert trainer offered 30–60-min 1-on-1 consultations for each trainee to (1) answer questions, (2) allow the local trainer to practice their slides for the upcoming booster, (3) provide individualized feedback on the local trainer’s presentation style, and (4) offer positive reinforcement and praise to increase confidence. Local trainers also received feedback during booster trainings from the expert trainer and their peers. All local trainers were deemed adequately prepared to move forward to lead their first training after actively participating in and completing all booster trainings*.*

#### Generation 2

Local trainers lead Generation 2 trainings independent of the expert trainer. For the first training led by each local trainer, a UC Berkeley facilitator attends to provide support with Zoom technology. The facilitator only answers content-related questions if requested by the local trainer. After the first training, facilitator support is offered but not required. Following conducting their first training, local trainers begin holding drop-in supervision hours to Generation 2 providers. Note, some local trainers preferred to hold regular supervision hours whereas others preferred to offer supervision on an as-needed basis, depending on trainers’ preference and scheduling capacity. Accordingly, local trainers also take on the responsibility of supervising cases on the path to TranS-C certification. Note, UC Berkeley facilitators continue to review submitted case materials and approve certifications. The expert trainer continues to hold drop-in consultation hours, open to both Generation 1 and 2 providers, and also holds individual consultation for the local trainers to support their transition to a supervisor role. During consultation for local trainers, the expert trainer clarifies advanced TranS-C content, consults on challenging TranS-C cases, and reinforces evidence-based supervision techniques. Additionally, the UC Berkeley facilitators host monthly “sleep expert network meetings” with all engaged local trainers, providing an informal opportunity for local trainers to learn from their new colleagues, build community, and discuss strategies to boost engagement in TranS-C among providers.

### Participants

Participants in the present study are drawn from CMHCs and consist of local trainers, Generation 2 providers, and Generation 2 patients.^1^ All participants are blind to condition (Standard vs. Adapted TranS-C), though are not blind to patient treatment allocation (immediate vs. delayed).

All CMHC sites from the Implementation Phase were invited to participate in the Train-the-Trainer Phase. The inclusion criteria for selecting the CMHC sites for the Implementation Phase were as follows: (1) provision of publicly funded adult mental health outpatient services and (2) support from CMHC leadership.

The inclusion criteria for local trainers were as follows: (1) employed in participating CMHCs; (2) completed a Generation 1 TranS-C training (i.e., led by UC Berkeley expert trainers); and (3) volunteer to participate and formally consent to participate. In reality, most trainers had completed their TranS-C certification (including completing TranS-C with three patients) or were progressing toward TranS-C certification and actively delivering TranS-C to patients [24].

CMHCs determined eligibility for Generation 2 providers (e.g., case managers, nurses, psychiatrists, training department staff), because this mirrors their real-world practice of determining who acquires additional training. For some CMHCs, this involves mandating TranS-C training for all untrained staff, whereas in others, leadership advertises the opportunity and allows anyone who is interested to register. The other inclusion criteria for Generation 2 providers are as follows: (1) employed or able to deliver patient-facing services to patients within the CMHC; (2) interest in learning and delivering TranS-C; and (3) volunteer to participate and formally consent to participate.

The inclusion criteria for patients are as follows: (1) aged 18 years and older; (2) meet criteria for an SMI per self-report and confirmed by referring provider or administration of the Mini International Neuropsychiatric Interview (DSM-5, Version 7.0.0) by a licensed clinical social worker on the research team; (3) exhibit a sleep or circadian disturbance as determined by endorsing 4 (quite a bit) or 5 (very much), or the equivalent for reverse scored items, on one or more PROMIS-Sleep Disturbance questions [35, 36]; (4) guaranteed place to sleep for at least 2 months that is not a shelter; (5) receiving the standard of care for the SMI and consent to regular communications between the research team and provider; and (6) consent to access their medical record and participate in assessments.

Patients will be excluded if they meet any of the following criteria: (1) presence of an active and progressive physical illness or neurological degenerative disease that is directly related to the onset and course of the sleep and circadian problems, or making participation in the study unfeasible, as assessed by the Checklist of Medical Conditions and Symptoms on the Duke Structured Interview for Sleep Disorders [37] and clinical interview; (2) presence of substance abuse/dependence only if it makes participation in the study unfeasible; (3) current active intent or plan to commit suicide (those with suicidal ideation are eligible) only if it makes participation in the study unfeasible, or homicide risk; (4) night shift work for more than two nights per week in the past 3 months (i.e., regularly scheduled work from 12 a.m.–6 a.m.); or (5) pregnant or breastfeeding.

### Recruitment

#### Community mental health centers

Building the CMHC network that forms the basis for this study began in August 2013 with outreach by the Principal Investigator (AGH). Originally, eight counties—each generally consisting of three to 10 CMHC sites—agreed to participate in the Implementation Phase. At various stages of the study, we have continued to recruit new counties and new CMHC sites to maximize provider and patient sample size goals for the Implementation and Train-the-Trainer Phases. For instance, all counties that participated in the Implementation Phase were invited to participate in the Train-the-Trainer Phase. Most elected to continue participating in the Train-the-Trainer Phase, with the exception of one county. Thus, to account for the latter, another county (Lake County) was recruited for the Train-the-Trainer Phase. Sites in the following nine counties in California, USA, are currently participating in the Train-the-Trainer Phase: Alameda, Contra Costa, Kings, Monterey, Placer, Santa Cruz, Solano, Santa Clara, and Lake. Note that sites in San Luis Obispo are also participating but are operating as part of Monterey County.

#### Local trainers

The UC Berkeley facilitation team works collaboratively with CMHC leadership, management, and champions (i.e., providers actively engaged and spearheading the TranS-C program in CMHCs) to identify and approach potential local trainers for participation. Benefits of becoming a trainer are emphasized, including certification as a TranS-C trainer, free training in teaching and supervision techniques, and career development opportunities.

#### Generation 2 providers

Generation 2 provider recruitment is a joint effort by UC Berkeley facilitators, CMHC leadership, and local trainers. UC Berkeley facilitators meet with key CMHC leadership, who help to engage and recruit Generation 2 providers in their CMHC. Facilitators encourage local trainers to engage and recruit Generation 2 providers by describing the benefits of participating in the study during their TranS-C trainings. These benefits include possible improvement in sleep and mental health for patients, certification in TranS-C for providers, expert consultation from the UC Berkeley research team, hard copies of the treatment materials, enrollment prizes, and financial compensation received by participating patients. After TranS-C trainings, local trainers send weekly emails for 1 month that highlight each of these benefits and present other resources related to TranS-C, sleep, and mental health. Providers are also recruited through flyers posted in CMHCs, announcements at staff meetings, meetings organized by the facilitators, and appointments by leadership. Strategies to maintain relationships with providers and optimize data collection are ongoing by facilitators, including workshops and trainings, “enrollment challenges” and prizes (e.g., treatment-related books, magnets, t-shirts, mugs, gift cards), continuing education credits for participation, and distributing newsletters or other topical resources. UC Berkeley facilitators encouraged local trainers to take part in or lead these efforts whenever possible.

#### Generation 2 patients

Patients for Generation 2 providers are recruited through a variety of methods, based on each CMHC’s preference. These methods include the following: (1) posting fliers from the research team in waiting rooms and providers’ offices; (2) integrating a sleep screener into intake paperwork; (3) asking providers to screen patients on their caseload; and (4) encouraging word of mouth between patients. Potentially eligible patients are typically identified by their providers. With the patient’s consent, the provider contacts the facilitators, who connect the patient with the assessment team so that the patient can be formally evaluated for eligibility and enrolled in the study. After eligibility has been confirmed and consent to participate in the study has been given, the patient is matched to a CMHC TranS-C provider. Ideally, the TranS-C provider is the patient’s own provider (e.g., their case manager, nurse, physician). If this is not possible, an alternative provider is identified. Patient retention is maximized via collaborative efforts between the providers, facilitators, local trainers (e.g., via supervision), and the assessment team. Considerable efforts are made by the facilitators and assessors to answer questions and troubleshoot challenges (e.g., scheduling difficulties) to prevent attrition.

### Interventions

As described above, two variations of TranS-C are tested in this trial: Standard TranS-C and Adapted TranS-C. Both are delivered alongside the usual care offered by each CMHC. The control condition is usual care followed by delayed treatment with Adapted or Standard TranS-C (UC-DT). In the CMHCs, usual care consists of working with a service provider (e.g., psychologist, case manager, occupational therapist, psychiatrist, nurse practitioner) who provides direct mental health support from within their scope of practice. The patient might also be referred by that provider for other services as needed (e.g., healthcare, housing support, nutrition, vocational specialists, peer advocacy). Occasionally patients receive treatment from interdisciplinary or residential teams, meaning their services are coordinated across multiple service providers. Although most providers deliver TranS-C via individual sessions, some choose to deliver it in a group setting. Note that TranS-C was originally developed in English, then translated into Spanish about 4 months into data collection to expand access. The Spanish translation of TranS-C was subsequently offered by Spanish-speaking providers. The treatment conditions, along with the adaptation process for Adapted TranS-C, are described below. The modules that make up Standard and Adapted TranS-C are compared in Table 1 and described in detail in Sarfan et al. (2023) [24]. While the ordering of modules is broadly suggestive of the order of completion, Generation 2 providers are trained to be sensitive to the differences between patients as to which processes are key to maintaining their distress and to address these processes at an earlier stage of treatment.

**Table 1:** TranS-C Modules—Standard and Adapted

Cross-Cutting Modules				Treatment Modules	Standard Modules *(Adapted)*
Functional analysis^a^	Education^a^	Motivational enhancement^a^	Goal setting^a^	Regular Sleep–Wake Times^a^	Core Module 1a *(Core Module 1)*
				Wind-down Routine^a^	Core Module 1b *(Core Module 2)*
				Wake-up Routine^a^	Core Module 1c *(Core Module 3)*
				Improving Daytime Functioning^a^	Core Module 2 *(Core Module 4a)*
				Unhelpful Beliefs about Sleep	Core Module 3
				Improving Sleep Efficiency	Optional Module 1
				Reducing Time in Bed	Optional Module 2
				Delayed or Advanced Phase	Optional Module 3
				Reducing Sleep-Related Worry^a^	Optional Module 4 *(Optional Module)*
				CPAP Machine and Exposure	Optional Module 5
				Negotiating Complicated Environments	Optional Module 6
				Reducing Nightmares	Optional Module 7
				Maintaining Your Gains^a^	Core Module 4 *(Core Module 4b)*

*Note* ^a^modules included in Adapted TranS-C

#### Standard TranS-C

Standard TranS-C is delivered in 8 × 50-min weekly sessions and comprised of 4 *cross-cutting modules* featured in every session, 4 *core modules*, and 7 *optional modules* that are used based on clinical presentation, treatment goals, and provider case conceptualization [18]. Training for providers in the Standard TranS-C condition consists of a 1-day workshop (i.e., 6–8 h) or two, 3-h training blocks.

#### Adapted TranS-C

Adapted TranS-C is delivered in 4 × 20-min weekly sessions and comprised of the same four *cross-cutting* and *core modules* as in Standard TranS-C. Modifications include: (1) the *cross-cutting modules* are standardized across sessions and scripted to reduce preparation time and (2) the core modules are split up into five (rather than four) modules. Additionally, there is one *optional module* which can be integrated with the core modules, based on clinical presentation, treatment goals, and provider case conceptualization. Training for the Adapted TranS-C condition consists of four, 1-h workshops or two, 2-h workshops, based on CMHC preferences.

There have been calls for rigorous approaches to treatment adaptation [38]. In response, we grounded the process for adapting TranS-C in theory, data, and stakeholder input. As the overarching guide for the adaptation process, the REP framework was used [23]. See Sarfan et al. (2023) [24] for a detailed description of the adaptation process for Adapted TranS-C. In sum, during Phase 1 of REP (Pre-Condition), we established that (a) there is a need for effective, feasible EBPTs for SMI in CMHCs, (b) sleep and circadian functioning may represent a powerful target to help address this need, and (c) there is empirical support for TranS-C in CMHCs [20] (see “Introduction”). Additionally, we gathered stakeholder input on fit and packaging of the intervention [21, 30]. We also reviewed past data and identified the TranS-C treatment skills that were most utilized by patients with a utilization scale adapted from Gumport et al. (2019) [39]. Next, we considered TranS-C’s theoretical underpinnings and mechanisms of action [18, 40] from which we retained the core elements [41, 42]. After, we piloted Adapted TranS-C with 21 adults through the PI’s UC Berkeley research clinic (unpublished data). Informal feedback was solicited from providers and patients who participated in this pilot to further refine Adapted TranS-C. In Phase 2 of REP (Pre-Implementation), we customized the delivery of TranS-C training and treatment materials to the CMHC context based on the input from CMHC leadership, staff, and patients [21, 30]. Throughout REP Phases 1 and 2, following leading adaptation frameworks, we sought to ensure that Adapted TranS-C would be relevant to the broadest range of patients and to account for factors that impact implementation (e.g., resources required) [42–44]. The present trial will address the last two phases of REP—namely, Phases 3 (Implementation) and 4 (Maintenance and Evolution).

#### UC-DT

**Fig. 2:**
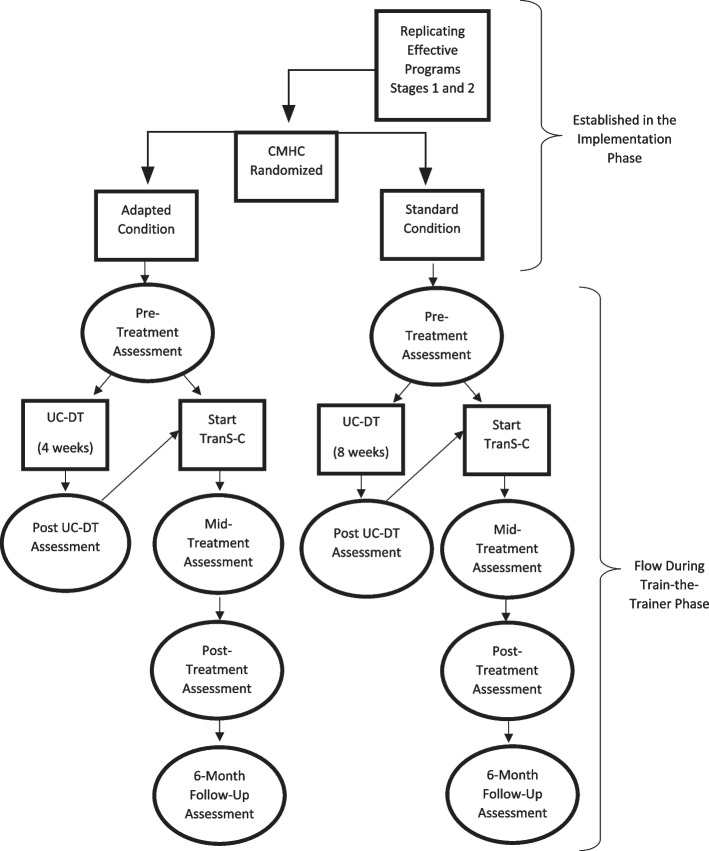
Community Mental Health Center (CMHC) Randomization and Patient Timeline

In UC-DT, patients begin with usual care for 4 weeks if their CMHC has been randomized to Adapted TranS-C or 8 weeks of usual care if their CMHC has been randomized to Standard TranS-C. After the delay, patients receive Adapted or Standard TranS-C, also based on the condition to which their CMHC has been randomized (see Fig. 2). The decision to compare TranS-C to UC-DT was made in close collaboration with the early CMHC partners. This design aims to strike a balance between (a) including a comparison group to demonstrate the effectiveness of TranS-C in community settings; (b) ensuring that *all* participants receive what we hypothesize to be an active treatment (TranS-C); and (c) maximizing efficiency in terms of study duration, budget, and participants’ time investment. Notably, usual care has been the comparison group in several influential studies (e.g., [45]).

### Measures

In addition to the measures below, a sociodemographics form is completed by providers and patients. Only measures that will be analyzed for the primary aims of the Train-the-Trainer Phase (see above) are reported below. See Table 2 for the timing of each measure.

**Table 2:** SPIRIT Depiction of Timing and Measures Collected for the Train-the-Trainer Phase

	**Screening**	**Post-Training**	**Pre-Treatment**	**Mid-Treatment**	**Weekly During Treatment**	**Post-Treatment**^**a**^	**6-Months Post-Treatment**
**Generation 2 Patient**							
Sociodemographics			x			x	x
Eligibility Items	x						
PROMIS-SD^b^	x		x	x		x	x
PROMIS-SRI			x			x	x
DSM-5 Cross-Cutting			x			x	x
SDS			x			x	x
Sleep Health Composite			x			x	x
PHENX Toolkit			x			x	x
CEQ						x	
**Generation 2 Provider**							
Sociodemographics		x					
Occupation		x					
Acceptability^b^		x				x	
Appropriateness		x				x	
Feasibility		x				x	
Weekly Session Log					x		

*Note*. Allocation to Adapted or Standard TranS-C occurs at the county level and prior to enrollment of any participants in that county (i.e., patients or providers). Enrollment of patients and allocation to immediate TranS-C or delayed TranS-C (UC-DT) occur after the screening and before the pre-treatment assessment. Enrollment of providers occurs after the training; note: providers may hold a dual role as a local trainer

^a^Post-treatment assessments for immediate TranS-C and delayed TranS-C (UC-DT) were identical except that the CEQ was not delivered at the UC-DT post-treatment assessment

^b^Primary Outcome

*PROMIS-SD *PROMIS-Sleep Disturbance; note: PROMIS-SD is only assessed during the pre-treatment assessment if done more than 1 month after the screening to minimize burden for patients, *PROMIS-SRI *PROMIS-Sleep-Related Impairment, *SDS *Sheehan Disability Scale, *CEQ*Credibility/Expectancy Questionnaire

#### Generation 2 providers

##### Primary outcome

***Acceptability.*** Generation 2 providers rate the acceptability of TranS-C via the *Acceptability of Intervention Measure* [46]. This 4-item measure is rated on a scale from 1 (completely disagree) to 5 (completely agree). This measure has demonstrated satisfactory known-group validity, internal reliability, test-retest reliability, and sensitivity to change [46].

##### Secondary outcomes

***Appropriateness and feasibility.*** Generation 2 providers rate the appropriateness and feasibility of TranS-C via the following 4-item measures: *Intervention Appropriateness Measure* and *Feasibility of Intervention Measure* [46]. Both measures are rated on a scale from 1 (completely disagree) to 5 (completely agree). These measures have demonstrated satisfactory known-group validity, internal reliability, test-retest reliability, and sensitivity to change [46].

##### Other measures

***Weekly session log.*** To assess the number of sessions delivered to each enrolled patient by each Generation 2 provider, providers complete a weekly survey in which they log each session for each patient.

***Occupation.*** Generation 2 providers are asked to report their current position, professional degree, and work history, including their caseload, theoretical orientation, licensure status, and previous training in sleep treatment.

#### Generation 2 patients

##### Primary outcome

***Sleep disturbance.*** The 8-item PROMIS-Sleep Disturbance (PROMIS-SD) assesses disruption to sleep (e.g., restlessness, trouble staying asleep) over the past 7 days [35]. Items are rated on a scale from 1 (not at all/never/very poor) to 5 (very much/always/very good), and scores range from 8 to 40, with higher scores indicating greater disturbance. This measure has demonstrated acceptable reliability and validity [35, 36].

##### Secondary outcomes

***Sleep-related impairment.*** The 16-item PROMIS-Sleep-Related Impairment (PROMIS-SRI) assesses daytime impairment related to sleep problems over the past 7 days on a scale from 1 (not at all/never) to 5 (very much/always) [35]. Scores range from 16 to 80, with higher scores indicating greater impairment (e.g., daytime sleepiness, difficulty concentrating). This measure has demonstrated excellent psychometric properties [35, 36].

***Functional impairment.*** Functional impairment is assessed via the Sheehan Disability Scale (SDS) [47]. Impairment in work and school, social life, and home and family is rated on a scale from 0 (not at all) to 10 (extremely). Scores range from 0 to 30, with higher scores indicating greater impairment. This measure has demonstrated good reliability and validity [47, 48].

***Overall sleep health.*** The Sleep Health Composite is proposed to capture overall sleep health for the complexity of sleep problems in SMI that are covered by TranS-C [49]. It is defined as the sum of scores on six sleep health dimensions (each dimension dichotomized as 1 = good; 0 = poor): Regularity (midpoint fluctuation), Timing (mean midpoint), Efficiency (sleep efficiency), Duration (total sleep time), Satisfaction (sleep quality question on PROMIS-SD), and Alertness (daytime sleepiness question on PROMIS-SRI). All dimensions—except Satisfaction and Alertness—are assessed via questions about sleep–wake patterns over the past 7 days (e.g., *In the past week, what time have you usually woken up in the morning?*). Scores range from 0 to 6, with higher scores indicating better sleep health. Initial validity of this measure has been established [49].

***Psychiatric symptoms.*** The DSM-5 Cross-Cutting Measure assesses psychiatric symptoms across 13 mental health domains. Participants rate how often they were bothered by each symptom on a scale from 0 (not at all) to 4 (nearly every day). Scores range from 0 to 52, with higher scores indicating more severe symptoms. This measure has demonstrated good test–retest reliability and clinical utility [50, 51].

##### Exploratory outcomes

***PhenX Toolkit: substance use and suicidality.*** Scales from the PhenX Toolkit [52] are used to assess various patient outcomes, including suicidal ideation and behaviors, alcohol, tobacco, and other psychoactive substances (e.g., cannabis, hallucinogens, sedatives). PhenX measures have been compiled by working groups and domain experts via a consensus process to facilitate consistency across studies [52]. To assess suicidal ideation and behaviors, the PhenX ‘Classification of Suicidal Ideation and Suicidal Behavior—Adult – Current’ protocol is used. This protocol includes two subscales from the screening version of the Columbia-Suicide Severity Rating Scale: Severity of Suicidal Ideation and Suicidal Behavior, assessing suicidality during two time periods—namely ideation in the past month and suicidal behavior in the past 3 months. To ease patient burden, this measure was adapted slightly, such that if patients deny suicidal ideation, they are not required to answer questions about suicidal behavior. To assess alcohol, the PhenX ‘Alcohol—30-Day Quantity and Frequency’ protocol is used. This protocol measures both quantity and frequency of alcohol consumption. To assess tobacco, the PhenX ‘Tobacco—30-Day Quantity and Frequency – Adult’ protocol is used. This measure has three sets of question protocols: (1) a protocol for ‘Every-Day Smokers,’ (2) a protocol for ‘Some-Day Smokers,’ and (3) a protocol for ‘Former Smokers.’ If patients report that they have never smoked tobacco, this measure is skipped. To assess use of substances and other drugs, the PhenX ‘Substances—30-Day Frequency’ protocol is used. This measure assesses use of substances such as sedatives, painkillers, stimulants, and hallucinogens. In addition, caffeine is assessed using questions adapted from the ‘Supplemental Beverage Questionnaire.’ Questions used in the present study assess frequency and quantity of caffeinated or decaffeinated drinks consumed over the past 30 days.

***Credibility and perceived improvement.*** Perceptions of TranS-C credibility and perceived symptom improvement are assessed by four questions adapted from the Credibility/Expectancy Questionnaire (CEQ) [53]. These questions assess (1) how logical TranS-C seemed, (2) how successful it was in reducing sleep symptoms, (3) how confident patients would be in recommending TranS-C to a friend, and (4) how much improvement patients believe had occurred. All questions are rated on a scale from 0 (not at all) to 9 (very), except for the final question (on perceived improvement), which is rated as a percentage from 0 to 100%.

### Procedure

Providers and patients are consented by the assessment team prior to participation. Although we do not collect trainer-specific data from local trainers, note that all trainers are required to complete a Generation 1 training, after which they provide consent to participate. All participants are informed that they can withdraw from the study at any time. All patients are compensated for their participation, and providers are compensated if permitted by their CMHC. Local trainers volunteer to become trainers and are not compensated; however, a certification in TranS-C training and a mug are provided if the trainer trains a minimum of 15 people across at least two trainings and supervises a minimum of three TranS-C cases*.*

Generation 2 provider and patient assessments are completed by the assessment team, comprised of experienced assessors. Note that assessors complete the consent process to minimize burden on participants. Because the assessors need to provide study-related information—such as number of assessments and treatment sessions—to the patients during the consent process, the assessors are not blind to condition at the pre-treatment assessment. However, at post-treatment and 6FU, we endeavor to keep assessors blind to condition. As is common in clinical trials, there are ways that assessors may be able to infer treatment condition (e.g., slightly different assessment batteries, patients may ask assessors “when does treatment start?” during the post-delay assessment). Assessors receive ongoing supervision and are thoroughly trained to deliver the surveys with integrity and minimal bias.

#### Local trainers

Trainers do not complete assessment batteries. Note that some trainers are also TranS-C providers in Generation 1 and complete the corresponding provider assessments (i.e., for Phase 1: Implementation Phase) [24].

#### Generation 2 providers

**Fig. 3:**
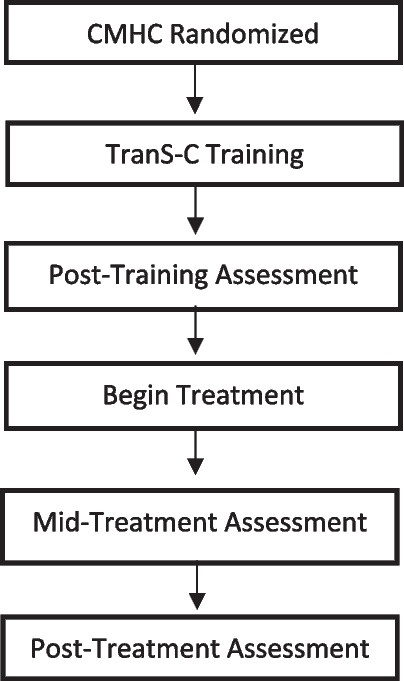
Generation 2 Provider Timeline. *CMHC = Community Mental Health Center

Provider assessments are completed after they complete TranS-C training, as well as at post-treatment. See Fig. 3 for provider timeline.

#### Generation 2 patients

Patient assessments in the immediate TranS-C treatment conditions are completed at pre-treatment, mid-treatment, post-treatment, and 6 months after treatment (6FU). Patient assessments in the UC-DT condition are completed at pre-treatment and 4 or 8 weeks after pre-treatment (i.e., post UC-DT), depending on whether their county has been randomized to Adapted or Standard TranS-C, respectively. After the post UC-DT assessment, patients start delayed treatment with TranS-C. They subsequently complete assessments at mid-treatment, post-treatment, and 6FU. Note that patients do not complete a 6FU assessment after the delayed portion of UC-DT. This was a compromise made with CMHC partners, so that patients would not need to wait 7–8 months to receive treatment. See Fig. 2 for patient timeline.

### Allocation

CMHCs and patients are randomized through a computerized randomization sequence. We do not stratify during randomization at the CMHC level. When randomizing patients, we stratify for presence of psychosis or not (current), presence of substance use or not (current), and age (≥ 50 or not), as there is evidence these variables can impact sleep or treatment outcome [30, 54]. Only the facilitators, assessors, and research team (i.e., not CMHCs, local trainers, providers, or patients) are privy to which CMHCs and patients are allocated to which TranS-C treatment condition (Adapted vs. Standard TranS-C). CMHC providers, local trainers, and patients know whether their patients have been randomized to receive the immediate or delayed treatment. The facilitator informs the local trainer once a patient can start having sessions, who then informs the provider. In the immediate condition, the provider is asked to begin sessions as soon as possible. In the delayed condition, the provider is asked to wait until after the patient has completed the post-delay assessment (i.e., approximately 4 weeks in the Adapted condition or 8 weeks in the Standard condition).

### Sample size

In the conceptualization of this study, the sample size goals for the Implementation Phase and the Train-the-Trainer Phase combined were 96 providers and 576 patients (including 20% for attrition). During the conduct of the Implementation Phase of the study, we realized the immense value to knowledge of both the Implementation Phase and the Train-the-Trainer Phase separately. Thus, we re-conceptualized the two phases as separate contributions. The Implementation Phase sample size remained as originally derived to power the analyses [24]. The sample size of the Train-the-Trainer Phase was guided by real-world factors, particularly the timeframe and budget for the study as well as the number of Generation 1 providers who are interested in recruiting, training, and supervising other providers. Additionally, in some CMHCs, many providers participated in Generation 1, leaving fewer providers to participate in Generation 2.

By the end of the Train-the-Trainer Phase, we project based on current recruitment numbers that we will recruit 130 patients and 60 providers. Using these sample sizes in a cluster-randomized trial design, minimum detectable effect sizes were calculated for Aims 1 and 2 using Stata [55] and Aim 3 using Schoemann et al.’s [56] application. For Aim 1, small to moderate correlations between TranS-C (vs. UC-DT) and sleep outcomes (*r*s = 0.37–0.39) and intraclass correlation (ICC) of 0.30 were estimated using data from a prior trial [20]. The coefficient of variation of cluster size was estimated as 0.72, based on the anticipated ratio of standard deviation of cluster size to mean cluster size for CMHC patients [57]. A two-sided alpha of 0.05 was used. Together, the minimum detectable effect size with a sample of 130 patients and 9 clusters was estimated at a large effect size of *d* = 0.94. We expect this effect size will be feasible to detect, given that a prior study with a similar aim and same primary outcome produced a similarly large effect size (*d* = 0.96) [20]. For Aim 2, prior studies have reported high sensitivity to change and test–retest reliability between measures of fit (rs = 0.83–0.85) [46]. Based on the ICC estimated from similar prior provider-level studies [23, 58], the ICC was assumed to be 0.20. The coefficient of variation of cluster size was estimated as 0.75, based on the anticipated ratio of standard deviation of cluster size to mean cluster size for providers [57]. A two-sided alpha of 0.05 was used. Together, the minimum detectable effect size with a sample of 60 providers and 9 clusters was estimated at a medium to large effect size of *d* = 0.70. Although few prior studies are available, one similar trial found a medium effect size (*d* = 0.53) [59]. Because these estimates suggest we might be slightly underpowered for Aim 2, effect sizes will be considered in addition to *p*-values. For Aim 3, a Monte Carlo power analysis through Schoemann et al.’s [56] application was conducted with 1000 replications, 20,000 Monte Carlo draws per replication, and 95% confidence intervals [56]. Drawing from prior research, medium correlations (*r* = 0.30) were assumed between the predictor (TranS-C condition) and mediators (acceptability, appropriateness, and feasibility, as well as mediators and outcomes (*r* = 0.50) [59]. Small correlations (*r* = 0.20) were assumed between the predictor and outcomes [60]. The power detected for the indirect effects with a sample size of *N* = 60 providers (i.e., for the mediators) was 0.62. As with Aim 2, because we may be underpowered to detect statistical significance at alpha = 0.05, effect sizes will be considered in addition to *p*-values.

### Data management and dissemination

All participant-identifiable data are saved by the assessment team on password-protected fillable PDFs on a secure password-protected and HIPAA-compliant website. On these PDFs, patients and providers are assigned identification numbers. Local trainers who entered the study as Generation 1 providers retain their original provider identification number. Local trainers who enter the study solely to be trainers are assigned an identification number. These identification numbers are then used to link anonymized data that is collected via password-protected Qualtrics. When collecting assessments, assessors call participants and enter the data into Qualtrics. Participants also have the option of entering their data directly into a participant-facing version of the surveys via a HIPAA-compliant version of Qualtrics. Participant-identifiable data is not shared with outside entities during or after the trial. A data management team supervised by the PI (AGH), biostatistician (LD), and postdoctoral scholar (LDS) is responsible for downloading, collating, and analyzing the data.

A Data Safety Monitoring Board has been formed to help prevent and manage adverse events. The board includes members with expertise in SMI, psychosocial treatments, and randomized controlled trials. Members are independent from the PI and competing interests. A report was made to the board bi-annually for the first year of the research of the Implementation Phase (Phase 1). Since then, it has shifted to annual reports. However, if safety issues arise, it will be changed to monthly meetings. Yearly reports are submitted to the Committee for the Protection of Human Subjects at UC Berkeley and National Institute of Mental Health (NIMH). Triyearly reports on recruitment are also submitted to the NIMH.

Organizations not directly involved in the trial (e.g., NIMH, Data Safety Monitoring Board, Institutional Review Board) have the right to audit and, if such a situation arises, will determine the frequency and procedures for auditing. The project management team regularly audits the monthly enrollment as well as the completeness and quality of the data.

Outcomes specifically of interest to our partners are presented to CMHC leadership as part of the widely used implementation strategy, audit, and feedback [61]. However, these interim analyses are used only for facilitation purposes and do not address the aims specified herein or by Sarfan et al. (2023) [24]. Also, they do not influence research procedures in any way (e.g., to inform when to terminate the trial).

Results from the trial, as well as analysis code, will be shared via peer-reviewed publications, professional conference presentations, and meetings and newsletters to CMHCs, as relevant. Other than the authors and compliance with data-sharing agreements stipulated by the National Institutes of Health, no other entities have contractual agreements to access the final dataset. Deidentified data are submitted to the National Institute of Mental Health Data Archive twice per year, per the NIMH requirements.

### Roles and responsibilities

This trial is supervised by the PI (AGH), who manages the facilitation team, assessment team, and the data management team. The PI meets with members of each team regularly and as needed in addition to daily email communication. Within each team, there is at least one trained lead (ERA, KF, JMS, LD, LDS) who supervises the day-to-day activities of other team members. There is no coordinating center, trial steering committee, or Stakeholder and Public Involvement Group. The responsibilities of each team are detailed elsewhere in this protocol. In summary, the facilitators execute the implementation of TranS-C via numerous activities, including training and supervising local trainers. The assessment team is responsible for the informed consent process and conducting participant (i.e., provider and patient) evaluations. CMHC leadership and enrolled local trainers and providers work with the facilitation team to recruit additional trainers, providers, and/or patients. Generation 2 providers help to identify potentially eligible patients, who are then connected with the assessment team for formal eligibility evaluation.

### Changes to preregistration

Originally, all phases of the trial were preregistered together on ClinicalTrials.gov on November 6, 2019 (identifier: NCT04154631). However, after much consideration, we decided to separate the three phases (i.e., Implementation, Train-The-Trainer, and Sustainment) in order to thoroughly investigate each phase, thereby maximizing research and partners’ resources, and contributing as much as possible to the field. Thus, on April 10, 2023, we created a separate ClinicalTrials.gov registration page for the Train-the-Trainer Phase (identifier: NCT05805657). This new page contains the information about the Train-the-Trainer Phase from the original preregistration but more thoroughly articulates the aims, hypotheses, measures, and procedures for this phase. After preregistration of the Train-the-Trainer Phase, we made one additional change. Specifically, given that change from pre-treatment to mid-treatment is not a primary outcome for any measure in the present study, we moved change from pre-treatment to mid-treatment on the Acceptability of Intervention Measure and the PROMIS-Sleep Disturbance measure from the primary outcome section to the secondary outcome section on ClinicalTrials.gov.

## Planned analyses

### Preliminary analyses and missing data

All analyses below pertain to the Train-the-Trainer Phase and are limited to Generation 2 providers and Generation 2 patients, except Exploratory Aim 1. For the primary analyses, which use multilevel modeling (MLM) and structural equation modeling, analyses will use all available data (intent-to-treat) [62]. Models will be estimated with maximum likelihood estimation, and missing data will be assumed to be missing at random [63]. For exploratory analyses using linear regression, approaches to missing data will be based on the number of missing cases (e.g., listwise deletion vs. multiple imputation) [64]. If dropout is related to other variables, they will be included as predictors. Baseline between-group differences in demographic variables will be examined. These tests will not be used to select covariates in the primary intention-to-treat analysis [65, 66]. Instead, covariates will be carefully selected at the conclusion of the trial—given the variations due to COVID-19 and the CMHC context (see “Discussion”)—and the potential influences of baseline differences will be evaluated as moderators (approach to moderation described below). Analyses comparing TranS-C to UC-DT will evaluate change in outcomes from pre-treatment to post-treatment. Analyses comparing Adapted to Standard TranS-C will evaluate change in Generation 2 outcomes from pre-treatment to post-treatment and pre-treatment to 6FU (see “Method” and “Discussion” for more details).

Distributions will be evaluated to detect outliers, and we will ensure that the assumptions of planned analyses are met. Covariates will include the patient variables for which we stratified (i.e., age and presence of psychosis or substance use). For all statistical models, counties will be adjusted for as a factor variable rather than a level of analysis due to the relatively small number of clusters. The average intraclass correlation on provider and patient-level outcomes will be reported.

### Dropout

The *N* by stage of dropout will be reported for the following: dropout after randomization but before the first treatment session, dropout after treatment has begun but attended half or fewer of the intended number of sessions (i.e., ≤ 2 in Adapted, ≤ 4 in Standard), dropout after attended more than half the intended number of sessions (i.e., > 2 in Adapted, > 4 in Standard) but before treatment has been completed, and dropout after treatment has been completed but prior to post-treatment or 6FU assessments. The number of Generation 2 patients who completed a post-treatment assessment but were lost to 6FU will also be reported. When available, the reasons for dropout and improvement among patients who drop out will be reported.

### Aim 1: Effectiveness outcomes of standard or adapted TranS-C versus UC-DT

Multilevel modeling (MLM) [63] will be used to account for multiple observations nested within patient. The level 1 equation will include dummy-coded time indicators as the predictor (0 = pre-treatment, 1 = post-treatment). The level 2 equation will include dummy-coded treatment condition (0 = UC-DT, 1 = Adapted or Standard TranS-C) and treatment by time interaction terms as predictors, adjusting for county. The treatment effects of interest will be significant treatment by time interactions at the 5% level on the primary outcome of sleep disturbance and the secondary outcomes of sleep-related impairment, functional impairment, and psychiatric symptoms, all modeled as continuous variables. Significant treatment by time interactions indicate that change in Generation 2 patient outcomes is significantly different comparing Adapted or Standard TranS-C to UC-DT. Significant interactions will be interpreted using planned contrasts (i.e., treatment effects on change from pre-treatment to post-treatment) and graphs. Additionally, the indirect effects of TranS-C relative to UC-DT on functional impairment and psychiatric symptoms through improvements in sleep disturbance and sleep-related impairment will be tested using multilevel structural equation modeling [67].

### Aim 2: Adapted TranS-C versus Standard TranS-C on fit to CMHC context

MLM will be used to account for multiple observations nested within Generation 2 providers. TranS-C treatment condition (Adapted vs. Standard TranS-C) will be evaluated as a predictor of fit, operationalized as Generation 2 provider ratings of acceptability, feasibility, and appropriateness. The level 1 equation will include dummy-coded time indicators as the predictor (0 = post-training, 1 = post-treatment). The level 2 equation will include dummy-coded treatment condition (0 = Standard TranS-C, 1 = Adapted TranS-C) and treatment by time interaction terms as predictors, adjusting for county. The treatment effects of interest will be significant treatment by time interactions at the 5% level on the primary outcome of acceptability and the secondary outcomes of feasibility and appropriateness, all modeled as continuous variables. Significant treatment by time interactions indicate that change in perceptions of fit is significantly different comparing Adapted to Standard TranS-C. Significant interactions will be interpreted using planned contrasts (i.e., treatment effects on change from pre-treatment to post-treatment) and graphs.

### Aim 3: Fit as a mediator of treatment condition and patient outcome

Multilevel structural equation modeling [67] will be used to test whether improved perceptions of fit (i.e., acceptability, appropriateness, and feasibility) mediate the relation between TranS-C treatment condition (i.e., Adapted vs. Standard TranS-C) and change in the primary patient outcome of sleep disturbance and the secondary patient outcomes of sleep-related impairment, functional impairment, and psychiatric symptoms in Generation 2. Models will evaluate change in outcomes from pre-treatment to post-treatment and pre-treatment to 6FU.

### Sensitivity analyses

Three sets of sensitivity analyses will be run to help account for the complexities of the COVID-19 pandemic and the CMHC context. In the first set, the analyses for Aims 1–3 will be conducted with (a) treatment completers, (b) patients who completed more than half the number of the suggested sessions (i.e., > 2 sessions for Adapted and > 4 sessions for Standard), and (c) patients who completed half or fewer of suggested sessions. In other words, these analyses will test the effectiveness of TranS-C at varying doses, which may be important considering evidence on “early responders” [68] and “real-world” contexts where turnover and dropout can be high [69]. In the second set of sensitivity analyses, we will assess whether any patients who did not complete post-treatment or 6FU had achieved meaningful clinical improvement by mid-treatment, using a reliable change index for the primary outcome of PROMIS-SD [70]. For the sensitivity analyses, we will define these patients (i.e., did not complete post-treatment or 6FU but achieved clinically meaningful improvement) as completers, and we will use their mid-treatment assessment in place of a post-treatment assessment. Then, all pre- to post-treatment analyses for Aims 1–3 will be rerun. In the third set of sensitivity analyses, we will run the analyses for Aims 1–3 but only include post-treatment and 6FU assessments that were collected within 3 months of the target assessment date (e.g., a 6FU assessment that was completed 9 months after treatment ended).

### Exploratory Aim 1: Generation 1 compared to Generation 2 on patient outcomes

To compare Generation 1 and Generation 2 on primary and secondary patient outcomes of TranS-C, data from the Implementation Phase (see Sarfan et al. (2023) [24]) and the Train-the-Trainer Phase and both TranS-C conditions (Adapted and Standard) will be combined. MLM will be used to evaluate interactions between generation (Generation 1 or Generation 2) and time. The level 1 equation will include dummy-coded time indicators as the predictors (0 = pre-treatment, 1 = post-treatment). The level 2 equation will include dummy-coded generation (0 = Generation 1, 1 = Generation 2) and treatment by time interaction terms as predictors, adjusting for county. A significant interaction indicates a moderating effect of generation and will be probed with planned contrasts (e.g., effects of generation on change from pre-treatment to post-treatment) and graphs.

### Exploratory Aim 2: TranS-C treatment condition on PhenX Toolkit and perceived credibility and improvement

MLM will be used to test TranS-C treatment condition (Adapted vs. Standard TranS-C) predicting change in PhenX Toolkit outcomes of substance use and suicidality from pre-treatment to post-treatment and pre-treatment to 6FU in Generation 2. The approach to MLM will mirror Aim 2, except the focus will be the Generation 2 patient data, outcomes will be substance use and suicidality from the PhenX Toolkit, and models will evaluate change from pre-treatment to 6FU. Linear regression will be used to test treatment condition (Adapted vs. Standard TranS-C) predicting patient perceptions of TranS-C’s credibility and perceived improvement at post-treatment.

### Exploratory Aim 3: Treatment effects moderated by risk factors

Using MLM, three-way interactions between treatment condition (Adapted or Standard TranS-C vs. UC-DT), time, and risk factors will be used to evaluate moderators of Generation 2 patient outcomes (i.e., sleep and circadian problems, functional impairment, and psychiatric symptoms). Each moderator and outcome will be tested in a separate model. Moderators will include age, sex, and sleep/circadian and psychiatric symptoms at baseline. The level 1 equation will include the moderator and dummy-coded time indicators as the predictors (0 = pre-treatment, 1 = post-treatment). The level 2 equation will include dummy-coded treatment condition (0 = UC-DT, 1 = Adapted or Standard TranS-C) and treatment by time by moderator interaction terms as predictors, adjusting for county. A significant interaction indicates a moderating effect and will be probed with planned contrasts (e.g., moderating effects on the differences between treatments in change from pre-treatment to post-treatment) and graphs. Simple slope analyses will be conducted for significant continuous moderators.

## Discussion

The second of a three-phase hybrid type 2 trial, the Train-the-Trainer Phase, aims to evaluate the implementation and effectiveness outcomes of the Transdiagnostic Intervention for Sleep and Circadian Dysfunction (TranS-C) delivered to patients in community mental health centers (CMHCs) by providers who are *trained and supervised within CMHCs* via train-the-trainer (TTT).

This novel study has the potential to make several significant contributions to the literature. First, to the best of our knowledge, this is the first study to utilize a TTT approach to implement sleep treatment for adults with serious mental illness (SMI) in CMHCs. Importantly, treating sleep and circadian dysfunction may be an efficient way to reduce the substantial societal burden of serious mental illness [20, 71] and few providers have received training in evidence-based sleep treatment [72]. Second, this study will assess the effectiveness of TranS-C in patients who are treated by Generation 2 providers (i.e., providers who were trained and supervised within CMHCs via TTT). As such, we will add to the growing body of work evaluating whether outcomes hold as training and supervision responsibilities transfer across generations of TTT. Third, this study evaluates two versions of TranS-C—Standard TranS-C and Adapted TranS-C—to determine if “fit” could be improved for Generation 2 providers. This is crucial as fit is an important predictor of implementation outcomes [25, 26], yet the impact of fit across generations of TTT is relatively unexplored. Evaluating this unique combination of implementation strategies—namely, TTT and treatment adaptation to improve fit to context—will contribute to the burgeoning evidence on causality in implementation science [73].

The potential contributions of this protocol must be considered alongside its methodological limitations. First, given real-world factors, particularly the COVID-19 pandemic, our sample size is unlikely to be optimal and analyses may be underpowered. That said, estimates of the minimum detectable effect size presented above suggest that the anticipated sample sizes for providers and patients may be sufficient for Aim 1 and we will use effect sizes in conjunction with *p*-values to interpret the findings for Aims 2 and 3. Additionally, relative to the existing TTT literature in CMHCs, the current estimated sample for both providers and patients is substantial [4]. Nonetheless, it is crucial for future research to examine TTT with larger samples to allow for more sophisticated analyses on the multitude of factors that may influence TTT outcomes.

Second, to reduce burden on our CMHC collaborators, we did not collect data from leadership nor local trainers at our partner CMHCs about their perceptions or feedback on TTT. We recognize that leadership-level factors can meaningfully impact implementation outcomes [74]. Therefore, exploring leadership perspectives on TTT and TranS-C is an important direction for future research. Additionally, very few studies have collected data from local trainers [14]. Thus, this area is ripe for both qualitative and quantitative research on a range of factors, such as local trainer attributes, effectiveness, competence, and perceptions of barriers and facilitators to sustaining the EBPT.

Third, as is common in TTT studies, our follow-up period is relatively brief (i.e., 6FU of TranS-C). It would be informative for future research to monitor outcomes over several generations of trainers and supervisors to provide robust tests of long-term TTT sustainability. Additionally, in the UC-DT condition, patients did not complete a 6FU assessment after the delay, and we did not collect 6FU data from providers. These were compromises made with CMHC partners to reduce burden and maximize participation in the study. Increasing our follow-up period to study the long-term effects of TranS-C relative to a control (e.g., UC-DT) and Generation 2 providers’ perceptions of TranS-C will be important directions for future research.

Fourth, flexible design choices were made at the facilitator, local trainer, and provider levels to respect the expertise and preferences of our CMHC partners. At the facilitator level, facilitation was scaffolded to meet the individualized needs of CMHC staff (including leadership, local trainers, and providers) in the transition to TTT*.* While standardized training procedures for local trainers were followed, evaluating the “readiness” of local trainers to lead trainings and supervisions was done on a case-by-case basis by the expert trainer, given the dearth of validated measures to guide this decision-making process. The development of optimal strategies for evaluating readiness and supporting trainers and supervisors in developing necessary skills for TTT should be a focus of future research [9]. At the local trainer level, individual trainers selected their preferred format for supervision of Generation 2 providers (e.g., group vs. individual, regular vs. as needed). At the provider level, providers had the option to deliver TranS-C in a group or individual format [24]. Collectively, these choices reflect the real-world restrictions and preferences of CMHC partners but introduced variance into the study. However, it has been critical to the CMHCs, local trainers, providers, and patients that we balance rigor with flexibility. At the conclusion of the trial, the sources of variation that resulted from the needs/preferences of our community partners will be carefully considered as to whether they should be included as covariates. Future research with larger sample sizes could consider standardizing and/or randomizing these variables to examine variations in TTT and identify any “tipping points” beyond which fidelity and clinical outcomes are undermined [9].

Finally, another potential limitation is sampling effects. The CMHCs, providers, local trainers, and patients who agreed to participate may not be representative of community mental health in general (e.g., with respect to perceptions of EBPTs, prior training in EBPTs). Nevertheless, a core strength of this study is that it is located entirely within practicing community mental health centers, and TranS-C is delivered as part of routine practice. In other words, this study takes steps toward ecological validity and support for effectiveness.

In sum, this study has the potential to (a) train a large number of CMHC providers and embed local trainers and supervisors to expand delivery of a promising transdiagnostic treatment for sleep and circadian dysfunction, (b) add to the growing body of TTT literature by evaluating Generation 2 outcomes with a novel treatment and population, and (c) advance our understanding of providers’ perceptions of EBPT “fit” across generations of TTT. Together, this study takes important steps toward testing implementation strategies (i.e., TTT and treatment adaptation) that have the potential to meaningfully impact the scale-up and sustainability of EBPTs.

## Trial status

Protocol version 1, May 11th, 2023. Data collection for the Train-the-Trainer Phase started in December 2020. Recruitment for the Train-the-Trainer Phase started in December 2020. Patient and provider assessments will continue through August 2023. Publishing of this protocol was delayed because of unforeseen challenges and uncertainties related to the COVID-19 pandemic and subsequent mandates (e.g., shelter-in-place), which began in California shortly after data collection started for this study. Also, during the Implementation Phase, we realized the immense value to knowledge of both the Implementation Phase and the Train-the-Trainer Phase separately. Thus, we re-conceptualized the two phases as separate contributions.

## Data Availability

Other than the authors and compliance with data-sharing agreements stipulated by the National Institutes of Health, no other entities have contractual agreements to access the final dataset. Deidentified data are submitted to the National Institute of Mental Health Data Archive twice per year, per their requirements.

## Abbreviations

TTT: Train-the-trainer
EBPT: Evidence-based psychological treatment
TranS-C: Transdiagnostic Intervention for Sleep and Circadian Dysfunction
UC-DT: Usual care followed by delayed treatment with TranS-C
SMI: Serious mental illness
CMHC: Community mental health center
PARiHS: Promoting Action on Research Implementation in Health Services
PROMIS-SD: PROMIS-Sleep Disturbance
PROMIS-SRI: PROMIS-Sleep-Related Impairment
PI: Principal investigator
REP: Enhanced Replicating Effective Programs framework
ICC: Intraclass correlation
MLM: Multilevel modeling

## Footnotes

1 Note that the vast majority of providers and patients were employed by or seeking/receiving treatment at CMHCs. However, in very few isolated instances, providers and patients outside of CMHCs learned about the study (e.g., by word of mouth) and requested to participate. When the providers or patients otherwise met the criteria, they were permitted to participate and were matched with a CMHC patient or provider, respectively, by the facilitation team.
